# Phytochemical diversity and seasonality are associated with a critical transition in plant–herbivore network structure

**DOI:** 10.1002/ecy.70282

**Published:** 2026-01-16

**Authors:** Leandro G. Cosmo, Kate P. Maia, Paulo R. Guimarães, Martin Pareja

**Affiliations:** ^1^ Department of Evolutionary Biology and Environmental Studies University of Zürich Zürich Switzerland; ^2^ Departamento de Ecologia, Instituto de Biociências Universidade de São Paulo – USP São Paulo Brazil; ^3^ Departamento de Biologia Animal, Instituto de Biologia Universidade Estadual de Campinas – UNICAMP Campinas Brazil

**Keywords:** chemical ecology, chemodiversity, critical transitions, ecological networks, giant component, plant–insect interactions

## Abstract

Understanding critical transitions in ecological systems is fundamental for addressing various natural phenomena, from population outbreaks to sudden ecosystem collapses. Ecological interactions are key drivers of these transitions, and theory suggests that the networks formed by these interactions can undergo their own critical transition. By examining interactions between plant individuals and insect species in a tropical forest, we first identified a critical network structural transition between the rainy and dry seasons. Next, we showed that seasonal changes and the phytochemical diversity of plants are associated with this transition. Finally, we quantified the consequences of the critical transition, which significantly increases the number of pathways and the potential for cascading effects among plants and herbivores in the network. Our findings reveal that ecological networks can experience abrupt changes on shorter timescales than previously recognized, with profound implications for cascading effects and the impacts of human‐induced perturbations on the stability of ecological assemblages.

## INTRODUCTION

**FIGURE 1:**
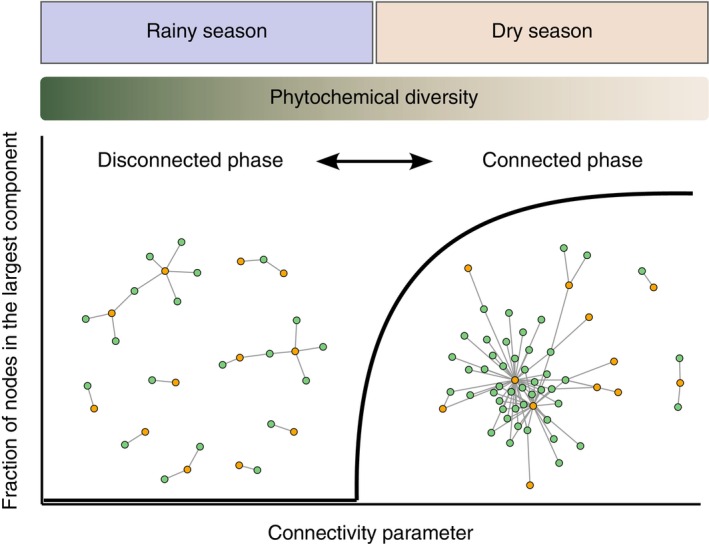
Theoretical representation of a critical transition and the different structural phases of a network. Networks can display two distinct structural phases: A disconnected phase with many components containing a small fraction of the nodes, and a connected phase in which most of the network nodes lie within a single component. Such a transition is predicted by a connectivity parameter (*C*, Equation 1), a quantity that is associated with the number and distribution of interactions in the system. We studied the network of interactions between *Piper amalago* individuals (green circles) and its herbivorous caterpillar species (orange circles) in the rainy and dry seasons. We hypothesized that the diversity of chemical compounds produced by *P. amalago* changes between seasons (represented by the changing colors in the bar labeled “Phytochemical diversity”). We further hypothesized that this transition between the rainy and dry seasons and the changes in phytochemical diversity are associated with structural changes in the network between a disconnected phase, where the network is fragmented into smaller components, and a connected phase, where the network contains a giant component.

Critical transitions—abrupt shifts in the qualitative state of a system—are widespread in ecology (Scheffer, 2020; Scheffer et al., 2012). From sudden populational explosions to the collapse of entire ecosystems, critical transitions may occur in many different ecological systems (Heffern et al., 2021). In these systems, previous research highlighted that ecological interactions are among the main drivers of critical transitions (Bascompte & Scheffer, 2023). Yet, theoretical models suggest that the networks formed by these interactions can undergo their own critical transitions. Specifically, the structure of networks can shift from a disconnected phase, characterized by isolated clusters of nodes (components), to a connected phase where most network nodes (e.g., more than 80%) are part of a single, extensive component known as the “giant component” (Figure 1; Guimarães, 2020; Newman et al., 2001).

Ecological networks can indeed exhibit various structures across the spectrum between the disconnected and connected phases (Guimarães, 2020). In other complex systems, the emergence of a giant component underlies the percolation thresholds of many phenomena, such as the spreading of epidemics or the efficiency and collapse of traffic and transportation networks (Buldyrev et al., 2010; Cohen et al., 2000; Xie et al., 2022). Similarly, in ecological networks, the emergence of a giant component may have an immediate consequence: it may not only increase the number of direct interactions among species but also abruptly increase the potential of species to indirectly affect each other. This increased potential for indirect effects occurs because of cascading effects—such as trophic, evolutionary, or extinction cascades—which can propagate freely through the indirect pathways connecting species within the same component but are disrupted when species belong to separate components (Cosmo et al., 2023; Guimarães, 2020; Guimarães et al., 2017; Higashi & Nakajima, 1995; Mittelman et al., 2022; Pires et al., 2020). However, the question remains whether a single ecological network can undergo a critical transition from disconnected to a connected phase, its ecological consequences, and what timescales might be involved.

Theory predicts that the emergence of a giant component depends on the distribution of interactions in the network. Specifically, a giant component may emerge when a network contains at least some highly connected nodes, whereas in fragmented networks all nodes interact only with a few others. In ecological communities, environmental factors can trigger changes in species traits, which in turn can suddenly reshape the distribution of interactions within networks. For instance, phenological traits, which determine the timing of life cycle events, can respond to short‐term fluctuations in temperature, precipitation, and resource availability, affecting the temporal overlap between interacting species and the structure of ecological interactions (CaraDonna et al., 2021; Olesen et al., 2008; Vázquez et al., 2023). Behavioral traits such as feeding preferences and foraging strategies can change with resource availability, modifying consumer‐resource interactions in food webs (Monteiro & Faria, 2018). Similarly, chemical traits, for example, secondary compounds in the leaves of plants, may vary with environmental stressors, affecting the palatability of plants to herbivores (Peralta et al., 2020). Consequently, ecological networks may transition from a disconnected to a connected phase depending on how traits respond to environmental variation and reshape interaction patterns.

Across different ecosystems, such response of traits to environmental variation—which can strongly affect the number and variance of interactions in ecological systems and mediate critical structural transitions—can occur over a short time scale. One such system is the one formed by plants and their associated herbivorous insect assemblages in tropical forests. On the one hand, many tropical forests alternate between two distinct seasons: a rainy season where both precipitation and temperature are favorable for growth and development, and a dry season where low (or even inexistent) precipitation can cause significant water stress and drastically reduce primary productivity, despite the relatively high temperatures (Alberton et al., 2023; Guan et al., 2015; Wagner et al., 2016). These seasonal changes are major drivers of the composition, abundance, and diet breadth of herbivorous insects, which in turn could drastically impact their patterns of interaction with plants (Cosmo et al., 2019; Dáttilo & Rico‐Gray, 2018; López‐Carretero et al., 2014; Morente‐López et al., 2018; Wolda, 1988, 1992). On the other hand, the diversity of chemical compounds in plant tissues (phytochemical diversity [PD]) has been shown to be a key trait mediating plant–herbivore interactions (Wetzel & Whitehead, 2020). PD shapes the interactions between plants and herbivorous insects across space, time, and different scales of organization, as PD can vary not only across plant species, but also among conspecific individuals (Cosmo et al., 2021b; Glassmire et al., 2016, 2019; Philbin et al., 2022; Richards et al., 2015). Therefore, PD could also be associated with abrupt transitions in the structure of plant–herbivore networks. Such critical transition to a more connected network, in turn, may both facilitate the propagation of disturbances, but also enhance community resilience (Thébault & Fontaine, 2010; Tylianakis et al., 2010). For instance, a more connected network can offer alternative host plants for herbivores when preferred hosts become unavailable or heavily defended, potentially preventing local extinction during resource‐limited periods (Staniczenko et al., 2010). From the perspective of plants, the increased connectivity may dilute herbivory pressure across more individuals, reducing the risk of high damage to any single plant (Barbosa et al., 2009). Unraveling whether the combined effects of environmental fluctuations and key traits, such as phytochemical diversity, can affect critical transitions in ecological networks is thus fundamental to our understanding of the processes underlying the structure and resilience of ecological communities over time.

Here, using the plant species *Piper amalago* L. and its herbivorous insects as a model system, we show that the network of interactions among plant individuals and herbivore species undergoes a critical transition from a disconnected structural phase in the rainy season to a connected phase in the dry season. This transition was associated with both the seasonality of tropical forests and the phytochemical diversity of plants. We further show that an immediate consequence of this phase transition is that insect species and plant individuals previously in separate isolated components suddenly become more susceptible to indirect and cascading effects through an increase in the number and importance of indirect pathways connecting plants and herbivores in the network.

## MATERIALS AND METHODS

### Individual plants and herbivore dataset

To perform this study, we used a dataset of the interactions of individuals of the neotropical shrub *P. amalago* (*n* = 124 individuals) and its herbivorous caterpillars (*n* = 18 caterpillar species) in two different seasons (dry and rainy seasons of 2017 and 2018) at the Reserva Biológica Municipal da Serra do Japi (Jundiaí, São Paulo, Brazil, 23°14′ S, 46°58′ W). The dataset is available in Cosmo et al. (2021a). *P. amalago* is a tropical perennial shrub whose leaves are rich in chemical compounds, and has caterpillars as its primary herbivores (Cosmo et al., 2019, 2021b). Briefly, the data were collected using the following sampling procedure. We sampled all of the naturally growing *P. amalago* plants along 6.7 km of trails at the study site. These plants were sampled at four different times over a year, two during the dry season (July 2017 and April 2018) and two during the rainy season (October 2017 and January 2018). In total, we sampled 62 individuals during the dry season and 62 during the rainy season, amounting to 124 individuals in total. From each plant, we manually collected all the caterpillars found. The collected caterpillars were identified as morphospecies in the field and then were taken and reared in plastic pots in a laboratory following standard procedures (Cosmo et al., 2014). Furthermore, we randomly collected 30 leaves per individual plant that were used to extract secondary metabolites and perform analysis of the phytochemical profiles of these plants. From this dataset, we used information on: (1) the two networks of herbivore species interacting with *P. amalago* individuals during the rainy and dry seasons (i.e., for each season, we aggregated data across years); (2) the frequency of caterpillar species on the host plants; (3) herbivory, expressed as the percentage of leaf area lost to chewing damage; and (4) the compositional and structural phytochemical diversity of secondary metabolites extracted and measured on *P. amalago* leaves (see below).

### Compositional and structural phytochemical diversity

Phytochemical mixtures can vary substantially in the richness and concentration of each compound, and this constitutes the compositional dimension of PD. Each compound, in turn, can vary in the composition and frequency of its chemical structures (e.g., a methyl or an amide group), which constitutes the structural dimension of PD (Cosmo et al., 2021b; Philbin et al., 2022; Wetzel & Whitehead, 2020). Therefore, the compositional dimension of PD is analogous to the species diversity in ecological communities, while the structural dimension is analogous to the functional (i.e., trait) diversity. Following previous work, we quantified both the compositional and structural PD of each leaf of each *P. amalago* individual using Shannon's entropy of the processed HPLC‐MS and 1H‐NMR spectra of methanolic extracts of *P. amalago* leaves, respectively (Glassmire et al., 2019; Philbin et al., 2022; Richards et al., 2015). Both the processed HPLC‐MS and ^1^H‐NMR spectra used in this study can be found in the dataset in Cosmo et al. (2021a).

### Network structural phases

Ecological networks can display structures corresponding to the two distinct structural phases described by random graph theory: a disconnected phase, where the network is a collection of isolated groups of interacting nodes (components); and a connected phase, formed by a giant component composed of most of the network's nodes (Guimarães, 2020; Newman et al., 2001). Graph theory predicts that the shift between the two structural phases occurs through a critical transition that is characterized by three main variables: (1) the number of components in each network; (2) the fraction of nodes in the largest component; and (3) a connectivity parameter that predicts the shift between the different phases for bipartite networks (Equation 1). In turn, the connectivity parameter that predicts the emergence of a giant component depends on the probability distribution of interactions in the network. Specifically, it depends on the average and the variance in the number of interactions of nodes in the network (degree). The higher the average and the variance in the degree of nodes, the more likely the network is to contain a giant component (Newman et al., 2001). Therefore, to explore whether ecological networks can undergo such a structural critical transition, we proceeded as follows.

First, to generate CIs for each of our measured variables (Casas et al., 2018), we used a bootstrap resampling approach and constructed 10,000 replicas of our network both in the dry and rainy seasons. To construct each replica, we randomly sampled, with replacement, interactions between individual plants and herbivore species from a given network, with probabilities proportional to the number of recorded interaction events, namely, to the frequency of each herbivore species in a plant individual. Thus, interactions involving frequent herbivore species were more likely to be sampled. The number of sampled interactions corresponded to the total number of interactions recorded in each empirical network. We also repeated this approach, varying the number of interactions sampled, which yielded the same qualitative results (Appendix S1).

Then, for each replica network we quantified three variables: (1) the number of components in the network; (2) the proportion of nodes (*P. amalago* individuals and herbivore species) in the largest component (in number of nodes); (3) the connectivity parameter that mediates the transition between the disconnected and connected structural phases (Equation 1) and, therefore, the presence of a giant component. For bipartite networks, which are composed of two sets of interacting nodes, as in plant–animal networks, this parameter is quantified as:

(1) $$C=\sum_{m=1}^{D_{\text{plants}}}\sum_{n=1}^{D_{\text{animals}}}\mathrm{mn}\left(\mathrm{mn}-m-n\right)p_{m}p_{n}$$

where *D*_plants_ and *D*_animals_ correspond to the largest degree observed in the set of plants and animals, respectively; *m* represents the degrees of nodes in the set of plants; *n* the degree of nodes in the set of animals; and *p*_*m*_ and *p*_*n*_ are the relative frequencies of nodes with degrees equal to *m* or *n*. The degree is the number of interactions of a given node, so that *m*, *n*, *p*_*m*_, and *p*_*n*_ together describe the distribution of interaction probabilities in the network (Newman et al., 2001).

Theory assuming fixed distribution of interactions per node and otherwise random interactions predicts that a critical transition occurs if the connectivity parameter is larger than zero (Newman et al., 2001). As we mentioned above, the connectivity parameter, and therefore the critical transition, are largely affected by the probability distribution of the number of interactions of nodes. While an even distribution of interactions among nodes is associated with a disconnected phase, an uneven distribution with a few highly connected nodes is associated with the emergence of a giant component and a connected phase. Hence, to gain insights into the processes that affect the structural phases of the plant–herbivore networks of the rainy and dry seasons, we used the relative frequency of interactions to calculate the probability of each plant *i* interacting with herbivores:

(2) $$\begin{array}{c} p_{i}=\frac{\sum_{j=1,i\neq j}^{N}w_{\mathrm{ij}}}{\sum_{i=1}^{N}\sum_{k=1,i\neq k}^{N}w_{\mathrm{ik}}} \end{array}$$

where *w*_*ij*_ is the frequency of herbivore species *j* that interacted with plant individual *i*. Note that $p_{i}$ quantifies the relative frequency of interactions of plants with herbivores and is different from herbivory, which was measured as the percentage of leaf area that each plant individual *i* lost to chewing damage.

### Statistical analysis

As discussed above, *p*_*i*_ is a main component within the connectivity parameter that predicts the transition between the disconnected and connected phases of networks. We used piecewise structural equation modeling (Lefcheck, 2016) to understand how seasonality, compositional and structural PD, and herbivory directly or indirectly are correlated with *p*_*i*_. Structural equation models provide a flexible framework for estimating hypothesized direct and indirect effects among variables, under the assumption that the model is correctly specified and the requisite causal assumptions (e.g., no omitted confounders) hold (Garrido et al., 2022). Based on previous work, we hypothesized the following causal relationships: (1) seasonality affects the compositional and structural PD; (2) both seasonality and compositional and structural PD affect the probability that plants interact with herbivores (*p*_*i*_, Equation 2); (3) compositional and structural PD affect herbivory. We also tested an additional structural equation model (SEM) including a path with induced chemical responses, which yielded a poorer fit than our initial hypotheses (Appendix S1). Model fit was tested with direct separation tests, which evaluate whether paths are missing in the model. All paths were fitted with linear models and Gaussian error distributions, except for the models with *p*_*i*_ as a response variable, which were fitted with a generalized linear model with binomial error distribution.

### Quantifying indirect pathways

When we proceed from a disconnected to a connected phase with a giant component, a major consequence can be the proliferation of indirect pathways. An example of indirect pathway in our plant–herbivore network is the indirect pathway of length 2 that connects two individual plants *i* and *j* that share a herbivore *k*. Networks can be represented by adjacency matrices, **A** *N* × *N*, in which *N* is the number of nodes and each element, $a_{\mathrm{ij}}$ = 1 if nodes *i* and *j* interact, and $a_{\mathrm{ij}}=0$ otherwise. For bipartite networks, this adjacency matrix can be built from the incidence matrix, **B**. The incidence matrix of a bipartite network with *N*_plants_ plants in set 1 and *N*_animals_ animals in set 2 is a matrix with dimensions *N*_plants_ × *N*_animals_ where each element *b*_*ij*_ represents the interactions between nodes belonging to different sets. Thus, the adjacency matrix, **A**, with dimensions *N* × *N*, has a block structure as follows:

$$A=\left(\begin{array}{cc} 0 & B\\ B^{T} & 0 \end{array}\right),$$

where the two blocks of zeros correspond to interactions among plants, with dimensions *N*_plants_ × *N*_plants_, and to interactions between animals, with dimensions *N*_animals_ × *N*_animals_; and $B^{T}$ is the transpose of the incidence matrix **B**.

In networks, the number of pathways of a given length can be quantified by calculating the powers of its corresponding adjacency matrix. When we raise **A** to the second power, the elements $a_{\mathrm{ij}}^{\left(2\right)}$ of the resulting $A^{2}$ matrix correspond to the number of pathways of length 2 connecting nodes *i* and *j*. The same applies to $A^{3}$, whose entries contain information regarding the number of pathways of length 3 connecting each pair of nodes. From graph theory, these pathways are formally defined as a walk of length ℓ, since they can revisit nodes and links in the network. Therefore, hereafter our definition of a pathway is a walk of a length ℓ in the network if we assume that the effects of pathways decrease with pathway length, the summation of the powers of **A**, $\sum_{\ell =1}^{\infty }{\alpha A}^{\ell }$, converges to the matrix of total effects, **T** (Guimarães, 2020; Katz, 1953; Pires et al., 2020):

(3) $$\begin{array}{c} \sum_{\ell =1}^{\infty }{\alpha A}^{\ell }=T={\left(I-\alpha A\right)}^{-1} \end{array}$$

where **I** [*N*,*N*] is the identity matrix, and α is a decaying constant for pathways of increasing length ($0<\alpha <\frac{1}{\lambda }$, with λ being the largest eigenvalue of **A**). The parameter α in Equation (3) underlies the assumption and condition under which the matrix $T={\left(I-\alpha A\right)}^{-1}$ exists. Specifically, this matrix exists if the effect of a given pathway decays with its length. This is the condition that allows the power series $\sum_{\ell =1}^{\infty }{\alpha A}^{\ell }$ to converge to the result above. A relevant example of this assumption in ecology would be in a food web, when the energy transferred between species decays as we move up across trophic levels (Borrett et al., 2007; Guimarães, 2020). This parameter controls the rate of decay and the value we chose was the minimum value so that the power series $\sum_{\ell =1}^{\infty }{\alpha A}^{\ell }$ converge to the matrix $T={\left(I-\alpha A\right)}^{-1}$. Since the minimum value of α is given by $\alpha <\frac{1}{\lambda }$, we used $\alpha =\frac{1}{\lambda +0.01}$.

The entries of **T**, $t_{\mathrm{ij}}$, correspond to the total number of pathways of any length connecting nodes *i* and *j*. Since these pathways can revisit nodes and links in the network, quantifying them using the **T**‐matrix also includes repetitive effects that can revisit nodes. However, we opted for this approach since it has a straightforward connection to the ecological dynamics of a system. For instance, in the traditional Lotka‐Volterra model, the steady‐state solution of abundances/biomasses can be written as ${\vec{N}}^{*}={\left(I-A\right)}^{-1}\vec{r}$ (Zelnik et al., 2024), where ${\vec{N}}^{*}$ is a vector of abundances/biomasses at the steady state, **A** is a matrix containing the pairwise direct effects of ecological interactions, and $\vec{r}$ is the vector of growth rates. Therefore, using Equation (3) to quantify number of pathways allows us to further interpret the results in light of the ecological dynamics of our system comprising plant individuals and herbivore species.

For each replica network we used its corresponding **T**‐matrix to quantify the relative contribution of indirect pathways to the total amount of pathways (Bonfim et al., 2023; Cosmo et al., 2023; Guimarães et al., 2017; Pires et al., 2020; Rother et al., 2025):

(4) $$\begin{array}{c} U=\frac{\sum_{i=1}^{N}\sum_{j=1,j\neq i}^{N}\left(1-a_{\mathrm{ij}}\right)t_{\mathrm{ij}}}{\sum_{i=1}^{N}\sum_{k=1,k\neq i}^{N}t_{\mathrm{ik}}} \end{array}$$

where $a_{\mathrm{ij}}=1$ if species *i* interact with species *j* or $a_{\mathrm{ij}}=0$ otherwise; and $t_{\mathrm{ij}}$ are the elements of the **T‐**matrix as defined above (Equation 3). This approach allowed us to understand how the contribution of indirect pathways changes as we move from the rainy to the dry season networks (empirical and replicas). These analyses were performed in the R environment v4.0.0.

## RESULTS

### Seasonality and the emergence of a giant component

**FIGURE 2:**
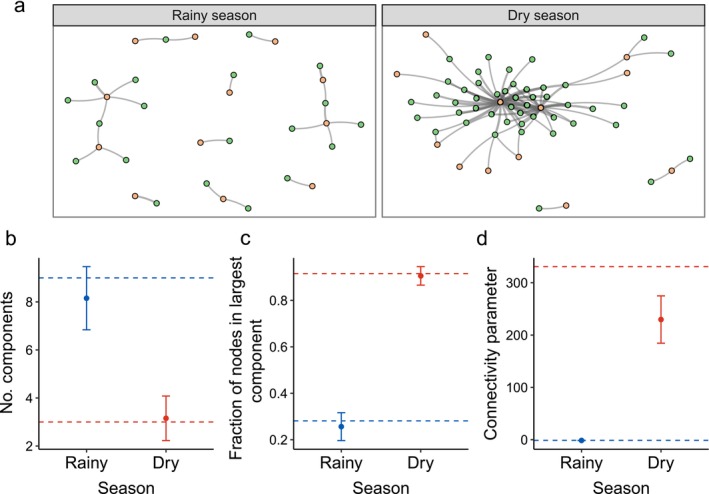
Transition from a disconnected to a connected structural phase from the rainy to the dry season in a tropical individual plant–herbivore network. (a) Visual representation of the networks of interactions between *Piper amalago* individuals (green circles) and its herbivorous caterpillar species (orange circles) in the rainy and dry seasons; (b) number of components in the network, (c) fraction of nodes in the largest component, and (d) value of the connectivity parameter (*C*, Equation 1) that predicts different structural phases in bipartite networks, for the rainy (blue points and lines) and dry seasons (red points and lines). In panels (b–d) points represent mean values and whiskers SDs for 10,000 bootstrap resamplings of each season's network. The dashed lines represent the value of the corresponding metric in the observed empirical networks.

Our first step was to describe how the structure of the studied plant–herbivore network changes from the dry to the rainy season in tropical forests. To do so, for each network, we quantified the number of components, the proportion of nodes in the largest component, and the connectivity parameter (Equation 1, *C*_rainy_ and *C*_dry_) that predicts the emergence of a giant component. We found that across seasons the network undergoes a critical transition (Figure 2a). In the rainy season, the network was divided into many components (8.17 ± 1.31 components, Figure 2b), the largest of which encompassed a small proportion of nodes (0.26 ± 0.06 nodes, Figure 2c) and, therefore, did not correspond to a giant component (*C*_rainy_ = −1.42 ± 0.18, Figure 2d). When the network proceeded to the dry season, it underwent a critical transition. In the dry season the plant–herbivore networks showed a small number of components (3.15 ± 0.92 components, mean ± SD, Figure 2b), the largest of which contained a high fraction of nodes (0.90 ± 0.04 in the largest component, Figure 2c) corresponding to a giant component (*C*_dry_ = 230.10 ± 46.75, Figure 2d). This critical transition was not a consequence of differences in network sizes, as in both seasons the network contained the same number of plant individuals and herbivore species (*n* = 62 plant individuals and *n* = 18 herbivore species).

### Structural and compositional PD have contrasting effects on the emergence of giant components

**FIGURE 3:**
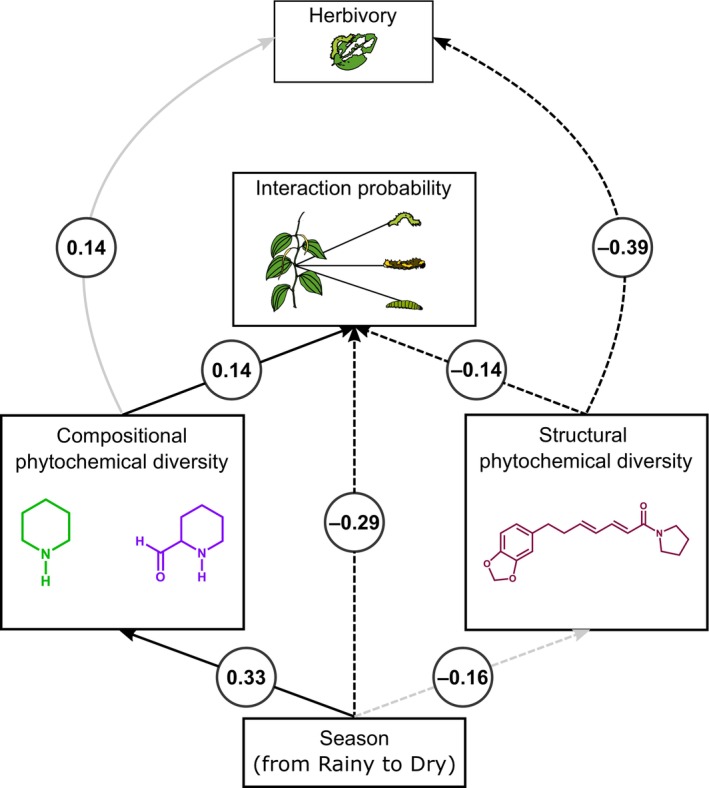
Results for the structural equation model (SEM) showing how seasonality, compositional phytochemical diversity (PD) and structural PD are associated with the probabilities of plants interacting with herbivores (*p*_*i*_, Equation 2), a condition for the emergence of a giant component in the network. Black lines correspond to statistically significant paths (gray lines otherwise). Solid lines denote positive effects, while dashed lines negative effects. All coefficients are standardized estimates. All of the plant, insect, and chemical compound illustrations are original and created by the corresponding author, Leandro G. Cosmo.

We next addressed the factors that correlate with the detected transition in the structure of the studied plant–herbivore network. Since the connectivity parameter that predicts the emergence of a giant component (*C*, Equation 1) depends on the probability distribution of interactions in the network, we used SEM to test how plant traits and seasonality could be correlated with the probability of plants interacting with herbivores (*p*_*i*_, Equation 2). Our results showed that the probability of plants interacting with herbivores shifted markedly between seasons. This shift was correlated with changes in the abundance distribution of caterpillar species from the rainy to the dry season. In particular, total caterpillar abundance increased from 29 to 244 individuals. The rise in abundance was mainly due to a sudden increase in the two most common caterpillar species found on *Piper* plants—both from the genus *Eois*—whose numbers increased from 7 and 1 individual to 170 and 59 individuals, respectively. As plant traits, we used phytochemical diversity and its different dimensions as this is known to be a key trait that mediates the interactions between plants and herbivorous insects. These different dimensions encompass a compositional one, that measures the diversity of chemical compounds, and a structural dimension that quantifies the diversity in the structural features that are the building blocks of each compound. The SEM showed that seasonality, structural PD and compositional PD all strongly affected the probability of individual plants interacting with herbivores (Figure 3, Fisher's *C* = 5.03, *p* = 0.28, df = 4, pseudo‐*R* ^2^ = 0.59), a property associated with the emergence of a giant component. The passage from the rainy to the dry season had a direct and positive effect on compositional PD (β = 0.33) and a direct negative effect on the plants' interaction probabilities ($p_{i}$, β = −0.29). Because compositional PD also had a direct and positive effect on $p_{i}$ (β = 0.14), seasonality had an indirect positive effect on $p_{i}$ by increasing compositional PD (β = 0.33 × 0.14 = 0.05). In contrast to compositional PD, structural PD had a direct negative effect on $p_{i}$ (β = −0.14) and herbivory (β = −0.39). Furthermore, structural and compositional PD were uncorrelated (Appendix S1: Table S1).

### Giant components and indirect pathways

**FIGURE 4:**
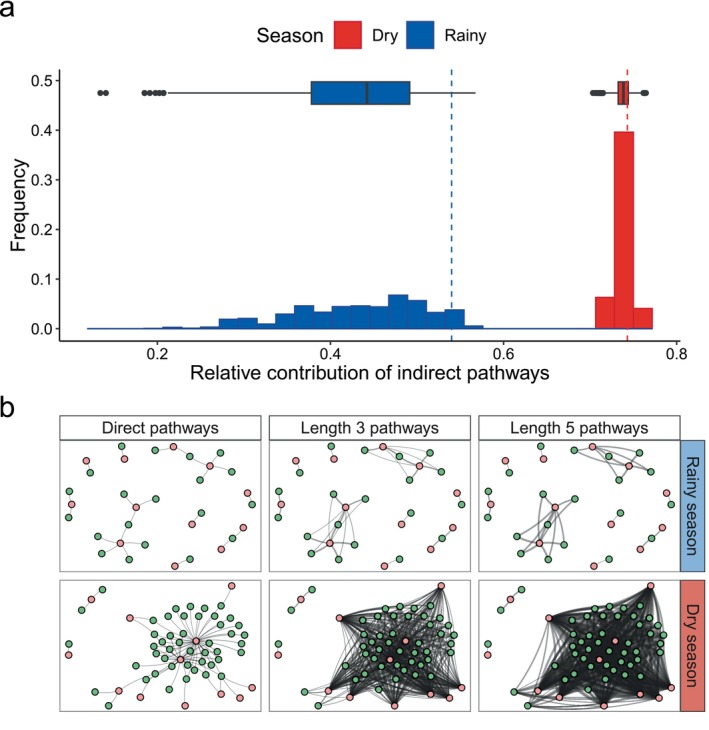
The contribution of indirect pathways increases from the rainy to the dry season in a tropical plant–herbivore network. (a) Histogram and boxplot showing the relative contribution of indirect pathways, in respect to the total number of pathways, in the rainy (blue) and dry (red) seasons networks. The dashed lines represent the contribution of indirect pathways in the observed empirical networks. (b) The increased contribution of indirect pathways is a consequence of the faster pathway proliferation that occurs in the dry season networks, relative to the rainy season. In the networks, herbivore species (orange circles) are linked to plant individuals (green circles) if there is at least one pathway of a given length that connects them. The width of the links between plant individuals and herbivore species represents the natural logarithm of the number of pathways of a given length connecting them (larger widths represent higher number of pathways). For simplicity only pathways of lengths 1, 3, and 5 are shown.

Finally, we quantified the potential consequences of the phase transition on how cascading effects spread in the studied system. In ecological networks, cascading effects depend on the number of direct and indirect pathways connecting species (Borrett et al., 2007; Cosmo et al., 2023; Guimarães et al., 2017; Higashi & Nakajima, 1995; Pires et al., 2020). Thus, we quantified the number and contribution of direct and indirect pathways in the rainy and dry season networks to assess how the emergence of a giant component shaped the potential for cascading effects in these networks. We found that the contribution of indirect pathways increased when we proceeded from the rainy to the dry season networks (Figure 4). While in the rainy season the contribution of indirect pathways corresponded to 43% of total network pathways (0.43 ± 0.08), in the dry season their contribution increased by 1.72‐fold (0.74 ± 0.01, Figure 4a). Interestingly, the variance in the possible realization of networks and contribution of indirect pathways was also higher in the rainy season network (Figure 4a), a property displayed by graphs that are very close to the critical point of the phase transition to a giant component (Newman et al., 2001). The increased contribution of indirect pathways during the dry season was a consequence of the faster increase in the number of indirect pathways of various lengths in the dry season network when compared to the rainy season (Figure 4b). Our sensitivity analyses showed that these results hold when we increase or decrease the number of interactions sampled in the bootstrap so that the dry and rainy season's network total number of interaction events matched each other (Appendix S1).

## DISCUSSION

Describing and understanding the processes underlying critical transitions has been a long‐standing goal in the study of many different complex systems, from physical to social ones. In ecological systems, understanding critical transitions is essential for understanding biodiversity since these transitions can lead to undesired states, as occurs when there is abrupt ecosystem collapse, species extinction, and pest outbreaks (Baruah, 2022; Dakos & Bascompte, 2014; Lever et al., 2014; Ong & Vandermeer, 2023; Vandermeer & Perfecto, 2019). Here, we show that the structure of empirical interaction networks may themselves undergo their own transition. Using a network describing interactions between plant individuals and herbivore species as a model system, we show (1) that a critical transition can occur over a short time scale in ecological networks; (2) that the combined effects of seasonal changes in the environment and a key plant trait, phytochemical diversity, are correlated with this critical transition, which (3) has major implications for how indirect and cascading effects propagate throughout the network. Taken together, our results have three main implications that may apply across a diversity of ecological systems once we know how pervasive critical transitions in ecological networks are.

First, we show that environmental fluctuations are associated with critical transitions in ecological networks. These fluctuations are widespread in nature and are very strong in tropical forests, despite these often being regarded as stable environments. Tropical forests usually alternate between two different seasons throughout the year, a dry and a rainy season, where precipitation can vary by several orders of magnitude. This alternation in the abiotic environment also changes the biotic environment through changes in plant productivity and the composition and abundance of herbivorous insects (Kishimoto‐Yamada & Itioka, 2015; Wolda, 1988, 1992). Previous studies with plant–herbivore networks in tropical forests showed that during the warmer and rainier months, herbivores interact more evenly with plant species, while in colder and drier months the frequency of interaction with generalist herbivores increases and interaction evenness decreases (López‐Carretero et al., 2014; López‐Carretero, del‐Val, & Boege, 2018; López‐Carretero, Díaz‐Castelazo, et al., 2018). Here, we show that seasonality is further correlated with a critical change in how herbivores interact with different individual hosts of the same species: the network of interactions undergoes a phase transition, from a disconnected phase in the rainy season to a connected phase in the dry season. Therefore, our study suggests that critical transitions may also occur across different seasonal systems, a question that deserves further attention, especially in tropical environments.

Second, together with different environmental conditions, individual traits are associated with critical transitions in the structure of ecological networks. In a previous study, we showed that variation in chemical diversity within individuals of *P. amalago* can affect the abundance, richness, and diversity of herbivores (Cosmo et al., 2021a, 2021b). Here, focusing on the temporal dynamics of the network between *P. amalago* individuals and herbivore species, we show that phytochemical diversity can be a key trait associated with large and sudden changes in the structure of this network. PD is a plant trait that shapes plant–insect interactions, from patterns of interaction and herbivore diversity to the strength of herbivory (Cosmo et al., 2021b; Glassmire et al., 2016, 2019; Mertens et al., 2021; Philbin et al., 2022; Poelman et al., 2009; Richards et al., 2015). Our results show that two dimensions of PD had contrasting effects on the probability distribution of the interactions of plants with herbivores: while structural PD had a negative effect, compositional PD had a positive effect. This contrasting effect indicates that a larger number of phytochemical compounds increases the probability of different herbivore species interacting with plants, but these interacting probabilities decrease when these compounds are structurally diverse. Though we can only speculate as to the mechanisms behind this, it is possible that compositional diversity is acting toward increasing interaction probability through positive effects on how herbivores locate hosts and on host–plant recognition, while structural diversity is enhancing the plants' defensive responses, as predicted by the synergy hypothesis (Whitehead et al., 2021).

Although plant secondary metabolites can influence herbivore feeding behavior and community composition (Richards et al., 2015), herbivores can also induce changes in plant chemical defenses (Karban & Baldwin, 2007). Thus, the relationship between plant chemistry and herbivore community structure likely involves feedback loops operating across different temporal scales. Short‐term induced responses to herbivory may influence plant chemistry within a season, while longer term evolutionary processes may shape constitutive defense strategies across generations (Agrawal, 2011; Endara et al., 2017). Our study captures a snapshot of these relationships, and studies across multiple seasons could further disentangle the underlying pathways. Nevertheless, our results highlight that the interaction probability distributions of plant populations may depend on a balance between the structural and compositional phytochemical diversity of plant individuals. An even interaction probability distribution favors a disconnected structural phase, while an uneven distribution favors a connected structural phase (Guimarães, 2020; Newman et al., 2001). Consequently, our findings suggest that any process affecting the interaction probability distributions in populations can shift ecological networks between disconnected and connected structural phases and vice versa. Thus, evolution, coevolution, or changes via inducible chemical defenses of the different dimensions of phytochemical diversity with herbivores can mediate complex feedback with network structure (Andreazzi et al., 2017).

Third, we show that the transition between different structural phases shapes the contribution of indirect pathways connecting species in the network. While networks composed of isolated components limit interactions mostly to pairs, in connected networks all nodes can affect each other via indirect pathways. For the network of *P. amalago* individuals and its herbivorous caterpillars, the contribution of indirect pathways increased greatly going from the rainy to the dry season networks. Such an increase in the contribution of indirect pathways was not gradual, as one could expect when we increase the connectivity of a network. Rather, it was an abrupt change, from a very low potential for indirect interactions to a very high one. As a consequence, the effects of the critical transition on the potential for indirect interaction go beyond a quantitative increase in the connectance of the network. The critical transition represents a qualitative transformation of the network's structure. Before the transition, the network is fragmented into a collection of small, isolated components. In this state, indirect effects are constrained within these small groups of species. However, after the transition, most species and individuals become interconnected through longer pathways and the potential for indirect interactions massively increases. Previous work showed that in ecological networks, a larger contribution of indirect pathways favors trophic, trait‐based, evolutionary, or extinction cascades (Cosmo et al., 2023; de Andreazzi et al., 2018; Duchenne et al., 2021, 2022; Guimarães et al., 2017; Higashi & Nakajima, 1995; Mittelman et al., 2022; Pires et al., 2020). Here, we show that at the level of individual plants and herbivore species, the potential for different types of indirect interactions can massively increase in the dry season of tropical forests.

These indirect effects can be manifested in several ways. For instance, consider two *P. amalago* individuals (P1 and P2) that do not directly interact but share a herbivore species (H1). When H1 feeds on P1, it may induce chemical defenses in P1 that make it less palatable, causing H1 to increase its feeding on P2—this module topology is similar to apparent competition where P1 indirectly negatively affects P2 through their shared herbivore (Holt, 1977; Morris et al., 2004). Alternatively, if P1 produces high levels of chemical defenses that increase mortality of H1, this can indirectly benefit P2 by reducing herbivore pressure—a form of indirect facilitation (Barbosa et al., 2009). Similarly, two herbivore species (H1 and H2) that feed on the same plant individual (P1) can indirectly affect each other. If H1's feeding induces chemical defenses in P1 that are effective against H2 but not H1, then H1 indirectly negatively affects H2 (Kaplan & Denno, 2007). Conversely, if H1's feeding damages plant tissues in a way that makes them more accessible or more palatable to H2, then H1 indirectly facilitates H2 (Ohgushi, 2005; Soler et al., 2012). In the disconnected network structure of the rainy season, these indirect effects are limited to small, isolated groups of plants and herbivores (components). However, with the emergence of a giant component in the dry season, these indirect effects can propagate throughout most of the network, connecting previously isolated groups. For example, if herbivore species H1 increases its feeding on plant P1, inducing chemical defenses, H1 might shift to feeding on plant P2, which could then affect plant P3 through a shared herbivore H2, and so on. These chains of effects were not possible in the rainy season when the network was fragmented into isolated components. This is particularly relevant for seasonality because the critical transition to a giant component coincides with the period when plants experience greater resource limitation, potentially altering the impact of herbivores on plant fitness. Taken together, these results suggest that the sudden change of the contribution of indirect effects between seasons can have major ecological and evolutionary consequences. Understanding these consequences would be an important next step toward comprehending how plant–herbivore network resilience varies seasonally, which would inform more effective conservation and management strategies.

Our study shows that an ecological network can undergo a critical transition over a short time scale. Together, our findings highlight that such critical transitions are associated with changing environmental conditions and the traits of individuals. Specifically, we show that the often‐neglected seasonality in tropical forests has important ecological consequences, and that phytochemical diversity can be a key trait associated with fast and abrupt changes in the structure of plant–herbivore networks. We further show that these abrupt transitions modify the contribution of indirect pathways and, consequently, the propagation of ecological and evolutionary cascades in the network. Since climate change is causing greater contrasts in precipitation through droughts and extreme precipitation events, seasonal differences are likely to become more pronounced over the coming years (Seneviratne et al., 2021), and these critical transitions in interaction networks and their ecological consequences could become more dramatic. Our study therefore sheds further light on how these shifts in environmental conditions and the traits of individuals can shape cascading effects in natural systems.

## AUTHOR CONTRIBUTIONS

Leandro G. Cosmo and Martin Pareja designed the study. Leandro G. Cosmo conducted the analyses. Leandro G. Cosmo, Kate P. Maia, Paulo R. Guimarães, and Martin Pareja wrote the first draft of the manuscript. All authors contributed substantially to further revisions.

## CONFLICT OF INTEREST STATEMENT

The authors declare no conflicts of interest.

## Supporting information


Appendix S1.

## Data Availability

Data and code (Cosmo et al., 2025) are available in Zenodo at https://doi.org/10.5281/zenodo.17236449.
