# Supplementary material for: Phytochemical diversity and seasonality are associated with a critical transition in plant–herbivore network structure
Source: Ecology. 2026 Jan 16;107(1):e70282. doi: 10.1002/ecy.70282 (PMC12811071; doi:10.1002/ecy.70282)
Supplement: Supplementary file 1 — Appendix S1. [file ECY-107-e70282-s001.pdf]

Supporting Information

Appendix S1

Phytochemical diversity and seasonality are associated with a critical transition in plant–herbivore network structure

Leandro G. Cosmo, Kate P. Maia, Paulo R. Guimarães Jr., Martin Pareja

Ecology

Contents

S1 Sensitivity analysis to the number of interactions in the rainy and dry seasons networks 2

Figure S1 3

S2 Alternative structural equation model 4

Figure S2 5

S3 Critical transition across different months 5

S4 Structural equation model report tables 7

Table S1 7

Table S2 7

S1 Sensitivity analysis to the number of interactions in the rainy and dry seasons networks

Our results show that the rainy and dry seasons networks of the interactions among *P. amalago* individuals and herbivore species undergo a critical transition. This critical transition is characterized by three main variables: (1) the number of components in each network; (2) the fraction of nodes in the largest component; and (3) a connectivity parameter that predicts the shift between the different phases for bipartite networks. Although the number of plant individuals and herbivore species observed were the same between the different seasons, the abundance of herbivore species and frequency of interactions is smaller in the rainy season than in the dry season of the study site. Thus, we performed an additional set of analysis to test the robustness of our results to differences in the frequency of interactions between the two networks.

In the additional set of analysis, we repeated our bootstrap resampling approach varying the number of interactions sampled as follows. We first constructed 10000 replicas of the rainy and dry seasons networks. For each replica, we sampled with replacement interactions between individual plants and herbivore species from a given network. Each interaction was sampled with probability proportional to frequency of interactions between each herbivore species and a given plant individual. The total number of interactions sampled was the same for the dry and rainy season replica networks. Using these 10000 replicas of the networks of each season, we then computed

the mean and confidence intervals of the three metrics mentioned above. For the sensitivity analysis, we repeated this procedure varying the number of interactions sampled. The number of interactions sampled was progressively increased in steps of 5, within the interval [29, 244]. In this interval, the lower and upper limits were given by the number of interactions observed in the empirical rainy and dry season networks. The results of this analysis showed that the results reported in the main text are robust to differences in the number of interactions observed in each of the networks (Figure S1). Regardless of the number of interactions sampled, the dry season network always had a smaller number of components (Figure S1a) and a larger fraction of nodes in the largest component (Figure S1b). Furthermore, the connectivity parameter also correctly predicted the different structural phases of the networks, with the rainy season showing values below and near zero (the critical point), and the dry season network above zero (Figure S1c).

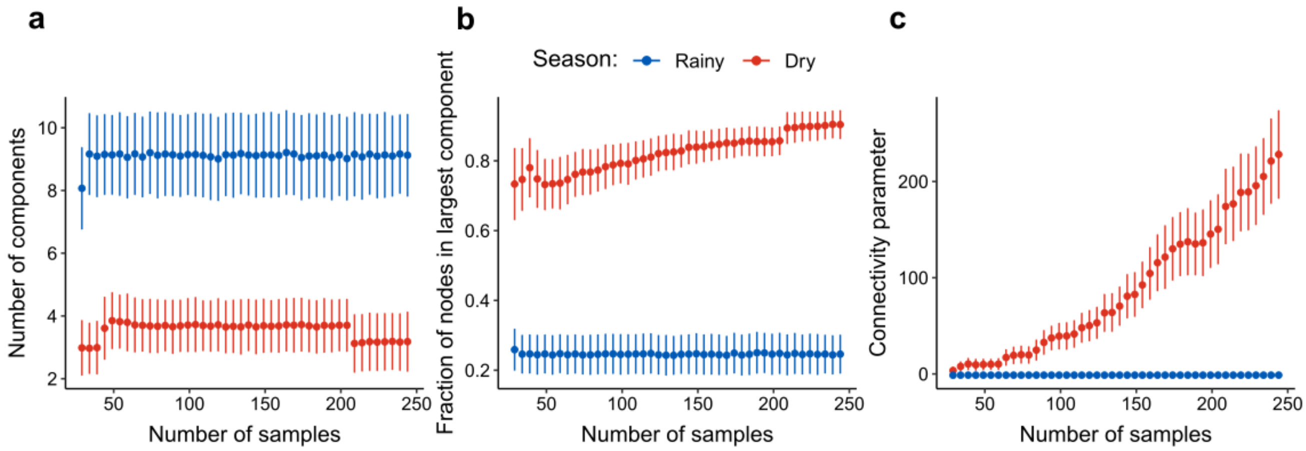

Figure S1 – Sensitivity analysis to the number of observed interactions of the transition from a disconnected to a connected structural phase in an individual plant-herbivore

network. (a) number of components in the network, (b) fraction of nodes in the largest component, and (c) the connectivity parameter predicting the structural phases in bipartite networks. In panels (a-c) points represent mean values and whiskers standard deviations for 10000 bootstrap resampling of the rainy and dry seasons networks.

S2 Alternative structural equation model

In the main text, we used structural equation modeling (SEM) to test our causal hypotheses about the variables associated with the probability of plants to interact with herbivores (our proxy for the critical transition). Our main causal hypothesis included the path in which the compositional and structural phytochemical diversity of plants affect the interactions probabilities of plants independent of herbivory. This causal path assumes that phytochemical diversity is not a product of plant induced responses to herbivores. Since induced responses could also be indirectly affecting the critical transition through its effects on phytochemical diversity, we tested an additional SEM in which we included the causal pathway corresponding to induced chemical responses (Figure S2). However, this additional SEM yielded a poorer fit than the model reported in the main text (ΔAICc $>$ 2, Tables S1-S2).

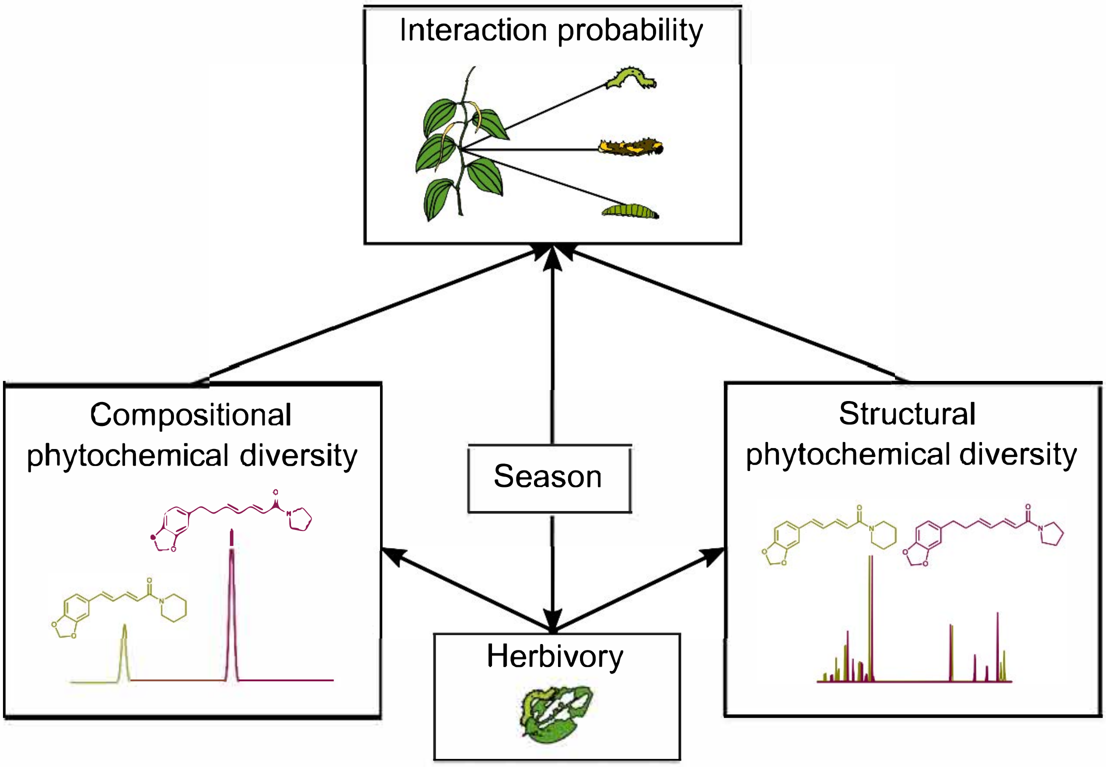

Figure S2 Causal hypotheses tested in the alternative structural equation model including a pathway corresponding to induced chemical responses. Arrows depict the direction of each causal hypothesis. All of the plant, insect and chemical compound illustrations are original and created by the corresponding author, Leandro G. Cosmo.

S3 Critical transition across years

In the main text we analyzed the critical transition in the structure of our empirical network between the rainy and dry seasons in a tropical forest. To do so, we pooled data of the interactions observed for four different months across the different seasons over the years of 2017 and 2018. Here, we test if the critical transition is also consistent across these two years. We found that, indeed, the critical transition in the structure of the network is consistent across years. Specifically, from the dry season of

2017 to the rainy season of 2017, the network transition from a single, giant component, to multiple components. This structure with multiple components persists into the second half of the rainy season, in January of 2018. After the rainy season, the network transitions once more to a giant component by the beginning of the dry season in April of 2018 (Figure S3).

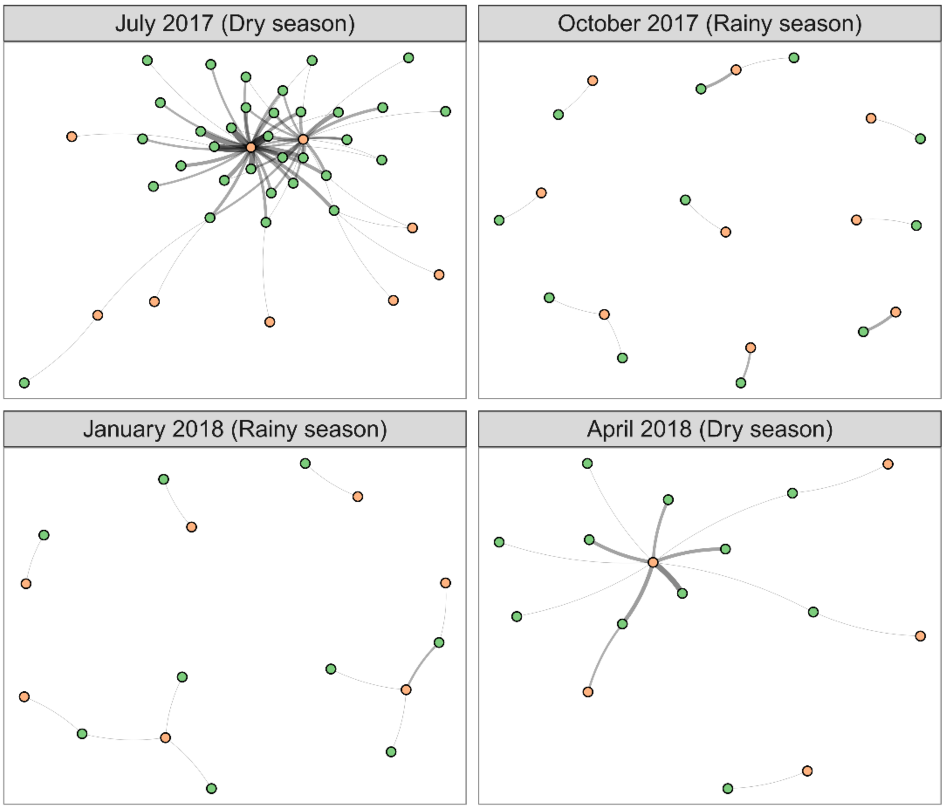

Figure S3 – Transition from a disconnected to a connected structural phase from the rainy to the dry season across two different years in a tropical individual plant-herbivore network. Each panel depicts a visual representation of the networks of interactions between *Piper amalago* individuals (orange circles) and its herbivorous caterpillar species (green circles) in the rainy and dry seasons of 2017 and 2018.

S4 Structural equation model report tables

Table S1 – Summary of the structural equation model used in the main text. The full names of the variables containing any abbreviation in the table are as follows: *PoI* – Probability of interaction of plants with herbivores; *Structural PD* – Structural phytochemical diversity; *Compositional PD* – Compositional phytochemical diversity. The effect of season corresponds to when going from the rainy to the dry season. Model AICc was 1290.714 and individuals R^2 for the response variables are as follows: *PoI* – $R^2 = 0.59$; *Structural PD* – $R^2 = 0.17$; *Compositional PD* – $R^2 = 0.04$; *Herbivory* – $R^2 = 0.02$.

Response	Predictor	Estimate	Std.Estimate	Std.Error	DF	P.Value
PoI	Structural PD	-0.015	-0.1452	0.003	63	<0.001 *
PoI	Compositional PD	0.1581	0.1399	0.0481	63	<0.001 *
PoI	Season	-1.1913	-0.2888	0.2111	63	<0.001*
Structural PD	Season	-6.2851	-0.1572	4.8982	65	0.204
Compositional PD	Season	1.2028	0.3296	0.4273	65	<0.01*
Herbivory	Compositional PD	0.0456	0.1386	0.0374	64	0.2278
Herbivory	Structural PD	-0.0117	-0.3911	0.0034	64	<0.001
~~Structural PD	~~Compositional PD	-0.1016	-0.1016	-	67	0.2084

Table S2 - Summary of the supplementary structural equation model including a path with induced chemical responses. The full names of the variables containing any abbreviation in the table are as follows: *PoI* – Probability of interaction of plants with herbivores; *Structural PD* – Structural phytochemical diversity; *Compositional PD* – Compositional phytochemical diversity. The effect of season corresponds to when going from the rainy to the dry season. Model AICc was 1295.675 and individuals R^2 for the response variables are as follows: *PoI* – $R^2 = 0.59$; *Structural PD* – $R^2 = 0.17$; *Compositional PD* – $R^2 = 0.04$; *Herbivory* – $R^2 = 0.02$.

Response	Predictor	Estimate	Std.Estimate	Std.Error	DF	P.Value
Herbivory	Season	0.1509	0.1258	0.1476	65	0.3106

PoI	Structural PD	-0.015	-0.1452	0.003	63	<0.001
PoI	Compositional PD	0.1581	0.1399	0.0481	63	<0.001
PoI	Season	-1.1913	-0.2888	0.2111	63	<0.001
Structural PD	Herbivory	-13.7126	-0.4114	3.768	65	<0.001
Compositional PD	Herbivory	0.596	0.196	0.3699	65	0.112
~~Structural PD	~~Compositional PD	-0.0738	-0.0738	-	67	0.278
